# Strategic 2-step surgery using thoracic endovascular aortic repair for an infected thoracic aortic aneurysm

**DOI:** 10.1093/icvts/ivac174

**Published:** 2022-06-30

**Authors:** Mitsunori Nakano, Atsushi Miyagawa, Daigo Shinoda, Koichi Yuri

**Affiliations:** Department of Cardiovascular Surgery, Tokyo Metropolitan Bokutoh Hospital, Tokyo, Japan; Department of Cardiovascular Surgery, Tokyo Metropolitan Bokutoh Hospital, Tokyo, Japan; Department of Cardiovascular Surgery, Tokyo Metropolitan Bokutoh Hospital, Tokyo, Japan; Department of Cardiovascular Surgery, Tokyo Metropolitan Bokutoh Hospital, Tokyo, Japan

**Keywords:** Infected thoracic aortic aneurysm, Thoracic endovascular aortic repair, Debridement, Two-step surgery

## Abstract

An 81-year-old man with multiple comorbidities developed infected thoracic aortic aneurysm, and we employed a strategic 2-step surgical approach combining thoracic endovascular aortic repair and local debridement with an omental flap during the active phase of infection. No signs of reinfection were observed at the 1-year follow-up. This strategy can be a safe and less invasive alternative to conventional open surgery in patients with high surgical risk.

## INTRODUCTION

Thoracic endovascular aortic repair (TEVAR) for infected thoracic aortic aneurysm (ITAA) has been implemented in patients with high surgical risk or as a bridge therapy to stabilize haemodynamic status. Whether local infection can be controlled after TEVAR without local debridement is a major concern. Here, we report a case of ITAA treated with a strategic 2-step surgical approach combining TEVAR and local debridement.

## CASE REPORT

An 81-year-old man with a history of hypertension, diabetes, chronic kidney disease and cerebral infarction presented to the emergency department with back pain. Laboratory data showed an elevated white blood cell count of 27 500/μl and a C-reactive protein value of 33.14 mg/dl. Contrast-enhanced computed tomography revealed a saccular aneurysm of the middle descending aorta (Video 1). A contained rupture of the ITAA was diagnosed.

**Figure 1:**
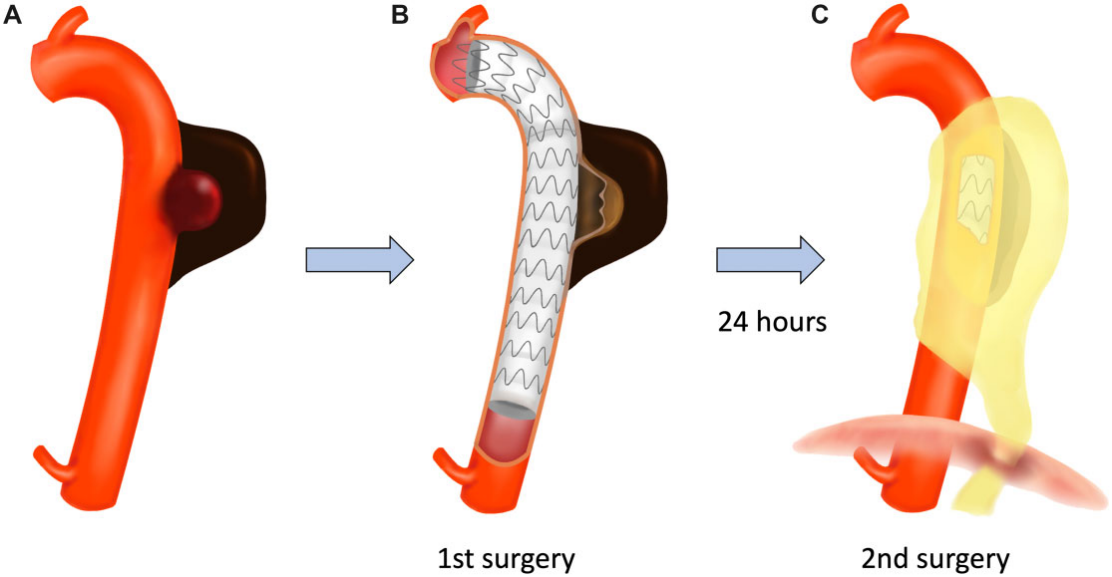
Schema of a 2-step surgical approach. (**A**) Preoperative image of infected thoracic aortic aneurysm. (**B**) Thoracic endovascular aortic repair. (**C**) Debridement and omental flap translocation.

*In situ* reconstruction using cardiopulmonary bypass was determined to be challenging owing to the patient’s general status. We planned a 2-step surgical approach using TEVAR (Fig. 1). Emergency TEVAR with a Conformable GORE TAG (CTAG) thoracic stent graft (Gore Medical, Flagstaff, AZ, USA) was performed (Video 1). Two stent grafts (proximal 31 × 200 mm, distal 28 × 150 mm) were deployed from the distal arch to the descending aorta at the level of Th11. After confirming no endoleaks by contrast-enhanced computed tomography, the second surgery was strategically scheduled on postoperative day 1.

**Figure 2:**
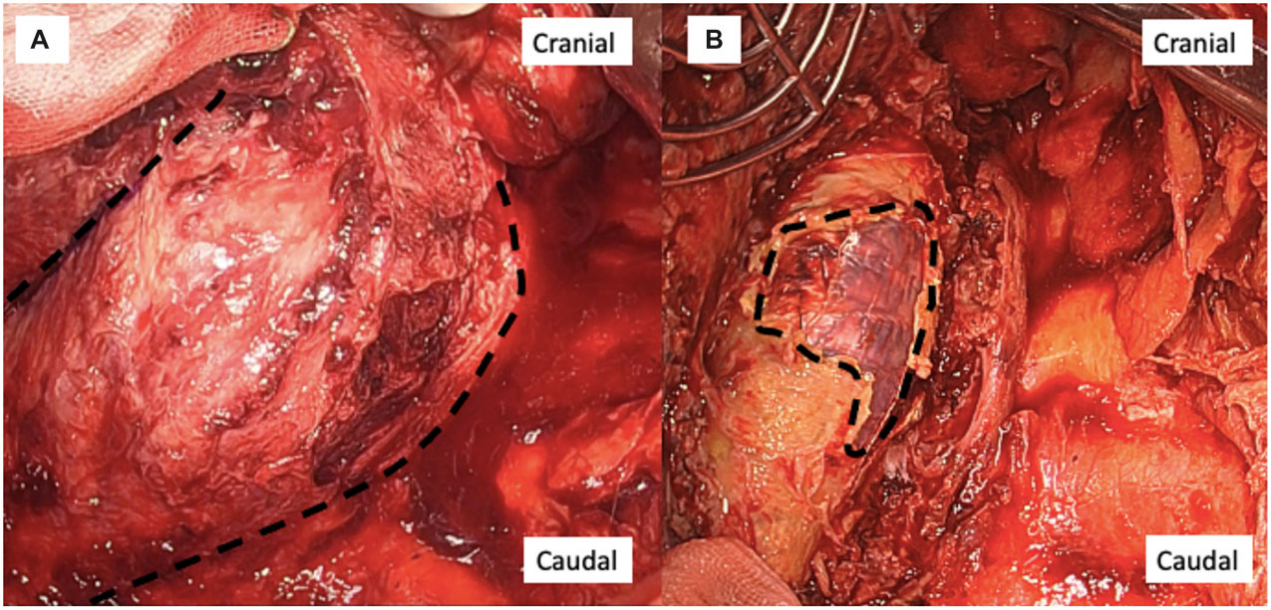
Intraoperative view. (**A**) Infected thoracic aortic aneurysm (dotted line) filled with purulent thrombus. (**B**) Stent grafts (dotted line) are partially exposed after the debridement of the aneurysm.

A left thoracotomy through the fifth intercostal space was performed. The dissection of the adhesion between the left lung and the aneurysm was carefully performed with thoracoscopic assistance. Purulent effusion oozed out from the aneurysm. Infected tissues, including the thrombus and aneurysmal wall, were resected. The stent grafts were partially exposed, but there was no bleeding from the surface (Fig. 2; Video 1). Blood leakage from the intercostal artery occurred and suture haemostasis was performed. The vascularized pedicled omental flap was translocated into the thoracic cavity through the diaphragm, which covered the descending aorta.

Methicillin-susceptible *Staphylococcus aureus* was identified in the blood culture on admission and in the intraoperative tissue cultures of the aneurysmal wall and thrombus. Antibiotic therapy was continued based on antibiotic susceptibility. Postoperative computed tomography revealed the descending aorta was covered with the omental flap without any abscess (Video 1). The patient was discharged on postoperative day 47. He received oral antibiotic treatment, and no signs of reinfection were observed at the 1-year follow-up.

## DISCUSSION

Some studies have reported acceptable long-term outcomes of endovascular treatment for infected aortic aneurysms. Sörelius *et al.* [1] reported the outcomes in 123 patients treated with endovascular aortic repair. A total of 33 patients (27%) developed infection-related complications, of whom 23 (70%) died. Eighty-two per cent of infection-related deaths occurred within 1 year. In the present case, we performed a prompt 2-step surgical approach during the active phase of the infection. The first step was emergency TEVAR to stop active bleeding and stabilize the haemodynamic status. The second step was local debridement to control the infection and prevent the spread of the infection through the stent grafts. Owing to the first step, the debridement was performed safely.

Some different approaches using TEVAR for ITAA have been reported. Kazuno *et al.* [2] reported the efficacy of TEVAR using pyoktanin (methylrosanilide chloride)-applied device. Pyoktanin has an antiseptic effect. They used a woven polyester graft device which has a risk of type IV endoleak. In the present case, we used a CTAG device made of expanded polytetrafluoroethylene that achieves zero porosity. No bleeding was observed from the surface of the stent grafts during the debridement. Hirano *et al.* [3] reported a case treated by a combination of TEVAR and video-assisted thoracoscopic debridement. They addressed the most important consideration is avoidance of blood leakage from the interstice between the aortic wall and the stent graft during debridement. In the present case, we encountered the blood leakage. Sufficient landing distance is important to prevent endoleaks and migration in suture haemostasis. This 2-step surgical approach can be a safe and less invasive alternative to conventional open surgery in patients with high surgical risk.

**Conflict of interest:** none declared.

## Notes

Presented at the 52nd Annual Meeting of the Japanese Society for Cardiovascular Surgery, Kanagawa, Japan, 3–5 March 2022.

## References

[1] Sörelius K, Mani K, Björck M, Sedivy P, Wahlgren CM, Taylor P et al; European MAA orators. Endovascular treatment of mycotic aortic aneurysms: a European multicenter study. Circulation 2014;130:2136–42. 25378548 10.1161/CIRCULATIONAHA.114.009481

[2] Kazuno K, Kinoshita H, Hori M, Yosizaki T, Tamura A, Sato H et al Endovascular treatment for mycotic aneurysm using pyoktanin-applied devices. CVIR Endovasc 2020;3:55. 32886250 10.1186/s42155-020-00151-0 PMC7474012

[3] Hirano K, Tokui T, Nakamura B, Inoue R, Inagaki M, Toyoshima H et al Hybrid therapy for mycotic aortic aneurysm with stent-graft and video-assisted thoracoscopic debridement. Ann Vasc Dis 2019;12:69–73. 30931062 10.3400/avd.cr.18-00119 PMC6434360

